# The combination of target motion and dynamic changes in context greatly enhance visual size illusions

**DOI:** 10.3389/fnhum.2022.959367

**Published:** 2022-09-15

**Authors:** Ryan E. B. Mruczek, Matthew Fanelli, Sean Kelly, Gideon P. Caplovitz

**Affiliations:** ^1^Department of Psychology, College of the Holy Cross, Worcester, MA, United States; ^2^Department of Psychology, University of Nevada, Reno, Reno, NV, United States

**Keywords:** visual perception, perceptual organization, dynamic illusion, size contrast, size constancy

## Abstract

Perceived size is a function of viewing distance, retinal images size, and various contextual cues such as linear perspective and the size and location of neighboring objects. Recently, we demonstrated that illusion magnitudes of classic visual size illusions may be greatly enhanced or reduced by adding dynamic elements. Specifically, a dynamic version of the Ebbinghaus illusion (classically considered a “size contrast” illusion) led to a greatly enhanced illusory effect, whereas a dynamic version of the Corridor illusion (a “size constancy” illusion) led to a greatly diminished illusory effect. Although these differences may arise from the different processes underlying these illusions (size contrast vs. size constancy), the dynamic variants we tested in our previous work also differed in the nature of the dynamic elements; specifically, whereas the Dynamic Ebbinghaus included a moving target and inducers that changed size and position, the Dynamic Corridor only included a moving target on a static background. Here, we explore further dynamic versions of the Ebbinghaus illusion and the Corridor and Ponzo illusions by separately manipulating three types of dynamic elements: target motion, context translation, and dynamic changes in context. Across five experiments examining 21 dynamic illusory configurations, adding target motion or a dynamically changing context separately resulted in little-to-no illusory effect. In contrast, the combination of target motion and a dynamically changing context led to a robust size illusion, consistent with an interactive effect. However, illusory effects that exceeded the matched classic, static illusory configuration were only observed for the dynamic versions of the Ebbinghaus illusion and the Revealed Ponzo illusions, in which the contextual elements changed size. We conclude that the combination of target motion and a dynamically changing context are necessary to produce dynamic size illusions, but that enhancement above and beyond static illusions may be largely specific to size contrast effects. Our results have important implications for the integration of motion signals, a ubiquitous environmental stimulus, in the perception of object size.

## Introduction

**FIGURE 1:**
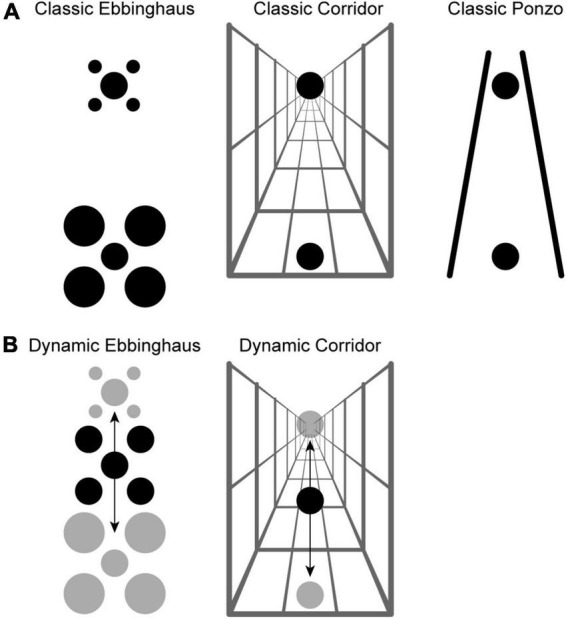
**(A)** Classic visual size illusions: Ebbinghaus, Corridor, and Ponzo. In all panels, the center black circles are all the same physical size. The surrounding context in each panel is such that the perceived size of the bottom black circle tends to be less than the perceived size of the top black circle. **(B)** Dynamic variants of the Ebbinghaus and Corridor illusion akin to those studied by Mruczek et al. (2020). The black arrow indicates the direction of motion of the stimulus. For the Dynamic Ebbinghaus, the entire stimulus (target and inducers) translated, and the inducers fluctuated between small and large. For the Dynamic Corridor, the target translated along a stationary corridor background.

The Ebbinghaus, Corridor, and Ponzo illusions (Figure 1A) are classic examples of a mismatch between the perceived size and the physical size of an object. In the Ebbinghaus illusion (Thiéry, 1896) a central circle surrounded by large-and-far inducers appears smaller than the same target surrounded by small-and-near inducers. The Ebbinghaus illusion is often described as a size contrast illusion (Mruczek et al., 2017b), although contour interactions between the target and inducers (Jaeger and Klahs, 2015; Todorović and Jovanović, 2018) and other factors (Rose and Bressan, 2002; Doherty et al., 2010; Kirsch and Kunde, 2021) also likely contribute to the illusory effect. In the Corridor illusion (von Bezold, 1884), which is most often described as a size constancy illusion (Yildiz et al., 2021), a circle placed at the “near” end of a corridor defined by pictorial depth cues appears smaller than the same circle placed at the “far” end of the corridor. Similarly, in the Ponzo illusion (Ponzo, 1912), converging lines around a pair of equally sized circles alters the perceived size of the circles through a combination of depth and contour interaction cues (Yildiz et al., 2021).

We have noted striking effects of motion dynamics on size contrast illusions. In the Dynamic Illusory Size Contrast effect (DISC) (Mruczek et al., 2014, 2017a), participants reported the perceived length of a black target bar surrounded by a white box. As in a typical size contrast effect, the perceived length of the bar was partially a function of the size of the surrounding box, with a larger box leading to a smaller perceived target length. Adding dynamic motion to the stimulus greatly enhanced the size illusion when both (1) there was target motion due to stimulus or eye movements and (2) the surrounding box dynamically changed size. Importantly, both of these factors were necessary to observe a strong illusory effect. In other words, the DISC effect is not due to target motion alone or a dynamic context, but the combination of both. We replicated the DISC effect using a dynamic version of the classic Ebbinghaus illusion (Figure 1B). By combining target motion, eye movements, changes in target eccentricity, and positional jitter, we observed an illusory effect for the Dynamic Ebbinghaus illusion that was almost four times as large as observed for the classic Static Ebbinghaus illusion (Mruczek et al., 2015).

In a recent follow-up study (Mruczek et al., 2020), we extended the exploration of motion dynamics on size illusions to the Corridor illusion, a classic size constancy illusion. In the Dynamic Corridor illusion (Figure 1B), a single target circle translated along a corridor background providing strong pictorial depth cues. Perhaps surprisingly, and at odds with neural models for the illusion (MacEvoy and Fitzpatrick, 2006; Ni et al., 2014), this target motion led to a much weaker illusion. The magnitude of the Dynamic Corridor illusion was approximately half that of the classic Static Corridor. One possible explanation for the opposite effects of motion dynamics on the magnitude of the Ebbinghaus and Corridor illusions is that motion dynamics affect size contrast and size constancy illusions differently. In our previous work, we raised the hypothesis that dynamic elements in the stimulus differentially influence the weights assigned to specific cues in the integrative processes that underly perceived size. However, an alternative explanation that we could not address with the dynamic variants we used in our previous study is that a dynamic context is key to inducing stronger size illusions when adding target motion. Indeed, as described above, our original study (Mruczek et al., 2014) suggested that both target and contextual dynamics were key factors for the DISC effect. In the Dynamic Ebbinghaus the inducers translated while growing and shrinking. In contrast, in the Dynamic Corridor configurations we previously tested, the background remained stationary, partially to avoid potentially disorienting effects by a global shift in the stimulus.

The experiments described in this manuscript were designed to test this latter hypothesis. Here we tested multiple dynamic versions of the Ebbinghaus, Corridor, and Ponzo illusions, which importantly included separate manipulations of target motion, context motion (i.e., translation), and dynamic changes in context (i.e., changes in the size or visible extent of the background context). We were particularly interested in whether multiple dynamic elements would lead to a Dynamic Corridor and Dynamic Ponzo illusion that was more robust relative to the classic, static illusions, as we have observed for the Dynamic Ebbinghaus. Across five experiments, we observed that dynamic versions of the Ebbinghaus, Corridor, and Ponzo illusion led to particularly robust illusion magnitudes when both target motion and a dynamically changing context were present, but not when only one of these factors was present.

## Experiment 1: Does a moving context enhance the corridor illusion?

Previously, we showed that adding motion dynamics to the Ebbinghaus illusion greatly enhances the illusion; in contrast, adding target motion to the Corridor illusion diminishes the illusion (Mruczek et al., 2020). One possibility for this discrepancy could be that while both the target and context are dynamic in the Dynamic Ebbinghaus configuration, in our previous work the target always translated against a stationary context in the Dynamic Corridor. The goal of Experiment 1 was to test a variant of the Dynamic Corridor illusion that included motion of the corridor context, in addition to simultaneous target motion. We also sought to replicate our previous observations of an enhanced illusion magnitude for the Dynamic Ebbinghaus illusion and a diminished magnitude for the Dynamic Corridor illusion with a stationary background.

### Materials and methods

#### Participants

Experiment 1 included 19 participants (nine female) including three authors. All participants reported normal or corrected-to-normal vision and all participants, except the authors, were naïve to the specific aims and designs of the experiments. Each participant signed an informed consent form before participating and was paid $8 upon completion of the experiment. All procedures were approved by the College of the Holy Cross Institutional Review Board before conducting any of the experiments.

To compute the expected statistical power of the experiments reported in this paper, we used G*Power (v3.1.9.4; Faul et al., 2007). Our previous studies of the effects of dynamic size illusions (Mruczek et al., 2014, 2015, 2020) have consistently revealed large effects sizes for significant pairwise comparisons. Using a conservative estimated effect size of *d* = 0.80 based on these past studies, a two-tailed test at α = 0.05 and a related-samples design with a sample size of 19 yields an expected power (1–β) of 0.91 for Experiment 1.

#### Display

Stimuli were generated and presented using the Psychophysics Toolbox (Brainard, 1997; Pelli, 1997; Kleiner et al., 2007) for MATLAB (2017b, The MathWorks Inc., Natick, MA, United States; RRID:SCR_001622). The experimental setup used a Dell UltraSharp 1908FP monitor (19 in, 1280 × 1024 pixel resolution, 60-Hz refresh rate) driven by a Mac Mini computer (2.6 GHz Intel Core i5, 8 GB of DDR3 SDRAM) with an Integrated Intel Iris 5100 graphics processor (1536 MB). Participants viewed the stimuli binocularly from ∼75 cm, without restraint. There were approximately 53 pixels per degree of visual angle.

#### Experimental design and conditions

**FIGURE 2:**
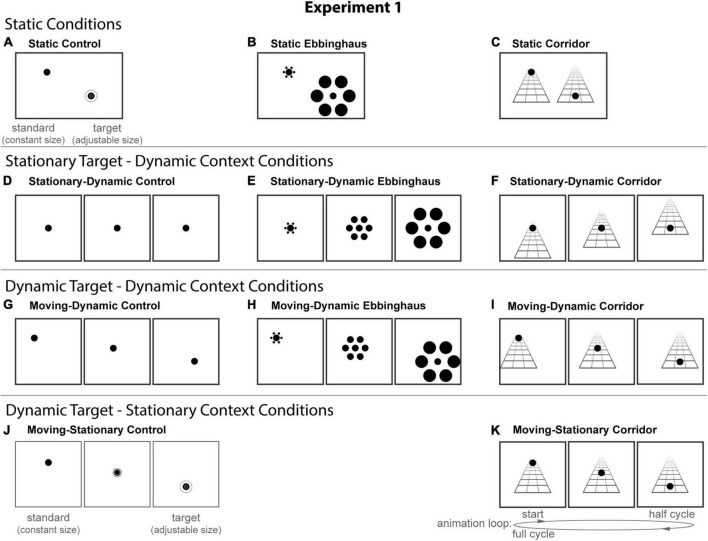
The 11 conditions of Experiment 1. Columns represent the three distinct contexts [Control **(A,D,G,J)**, Ebbinghaus **(B,E,H)**, Corridor **(C,F,I,K)**]. Rows represent the four configurations [Static **(A–C)**, Stationary-Dynamic **(D–F)**, Moving-Dynamic **(G–I)**, Moving-Stationary **(J,K)**]. We did not include a Moving-Stationary Ebbinghaus as it was not possible to create this an Ebbinghaus variant with target motion, but without context motion. For all Dynamic conditions **(D–K)**, screenshots for the beginning (standard circle), middle, and end (target circle) frames are shown. The animation [as labeled in panel **(K)**] played in a continuous loop. In the figure, the circle is the same physical size in all frames; during the experiment, the target (lower right circle for Static conditions or the far-right frame for Dynamic conditions) was adjusted by the participant [see panels **(A,J)**]. Here, stimuli are cropped to display the central portion of the screen to save space; stimulus components (e.g., Ebbinghaus inducers) did not come so close to the edge of the monitor. Supplementary Video 1 showing all of the dynamic conditions for Experiment 1 can be found in the Supplementary materials (best viewed in “loop” mode).

All stimuli were composed of black geometric shapes presented on a white background. Each participant was shown 11 distinct conditions (Figure 2) formed from crossing three contexts (Control, Ebbinghaus, and Corridor) and four configurations that manipulated the dynamics of the stimulus (Static, Stationary-Dynamic, Moving-Dynamic, and Moving-Stationary; labels refer to the target circle and surrounding context were dynamic using a “target motion-context dynamics” labeling convention). Supplementary Video 1 showing all dynamic conditions can be found in the Supplementary materials (best viewed in “loop” mode). For reasons outlined below, we did not include a Moving-Stationary Ebbinghaus condition, and thus do not have a complete factorial design. Seven participants completed 12 trials per condition for a total of 132 trials; the remaining participant completed 8 trials per condition for a total of 88 trials. Participants were allowed to take self-timed breaks after every 20% of completed trials.

The experiment used the method of adjustment to obtain a direct estimate of the participants point of subjective equality (PSE) (see below) on every trial. For the static conditions, participants moved a computer mouse up and down to adjust the size of a target circle to match the size of a nearby standard circle (illustrated in Figure 2A). In the dynamic conditions, participants adjusted the growth rate of a target circle until they perceived it as not changing in size over time (illustrated in Figure 2D). Depending on the context (Figure 2, columns), the target could be isolated (Control conditions), flanked by inducers (Ebbinghaus conditions) or overlaid on a static grid pattern (Corridor conditions). Participants pressed the spacebar to indicate when the size of the target appeared to match the standard (static conditions) or did not appear to change over the course of the trial (dynamic conditions). There were no time limits; participants could take as much time as needed to make their response. Participants were allowed to freely view the display. Although free viewing has the potential to create additional variability across participants, it is a conservative approach in that the extra time allotted to participants allows for strategies and adaptation to the stimulus that mitigate illusion magnitudes overall.

Below, we describe each of the 11 conditions and the behavioral task, starting with the three static conditions (Figure 2, top row), which replicate the classic illusion configurations. For all static conditions there were two circles: the standard and the target. Participants adjusted the target size to match the perceived size of the standard (illustrated in Figure 2A).

##### Static control

The static control condition (Figure 2A) was a simple size comparison between two isolated circles, with no additional context. The standard and target circles were placed diagonally opposed to each other. The standard was always located in the top left quadrant of the screen, −3.5° left and 4° above the center of the monitor. The standard width was either 1.8 or 2.0° (equal number of trials of each) and it remained the same size throughout the trial. The target was always located in the bottom right corner of the screen, 3.5° right and −3° below the center of the monitor. The width of the target was adjusted by the participant within the range of 0.2–5.75°. At the start of the trial, the target size was initialized to 10% (0.76°) or 90% (5.20°) of the range of possible sizes, with an equal number of trials initialized to the small or large target. Thus, on half of the trials, participants were initially required to increase the target size; on the other half of the trials, participants were initially required to decrease the target size. However, participants were free to increase or decrease the target size as many times as they liked until they made in their final response.

##### Static Ebbinghaus

The Static Ebbinghaus condition (Figure 2B) was the familiar Ebbinghaus configuration, in which the target and standard circles were surrounded by different sized inducers. In order to match the expected perceptual effects of the Corridor illusion (see below), the target (lower position) was always flanked by the large inducers and the standard (upper position) was always flanked by the smaller inducers. The target, whose size was adjusted by the participant, was surrounded by six equally spaced large inducers (3.75° width, 4.75° eccentricity). The standard was surrounded by six equally spaced small inducers (0.7° width, 1.5° eccentricity). The size and position of the standard was identical to the static control condition, as was the position of the target and the range of adjustable target sizes. Indeed, the maximum adjustable target size was set (across all conditions) such that the target could not overlap with the inducers.

##### Static corridor

For Experiment 1, the Static Corridor condition (Figure 2C) used a corridor background in which the walls were removed and only a grid floor was present. We used this simplified corridor context to minimize any disorienting global motion effect during translation of matched dynamic conditions. The grid floor covered a width of 11° at its base and a height of 9.625°. In the Static Corridor condition, the standard and target were presented overlayed on separate grid floors, with the standard being in the “far” (upper left) position and the target being in the “near” (lower right) position. These positions matched the locations for the Static Ebbinghaus and static control conditions. The size of the standard and the range of adjustable target sizes were also identical to the other static conditions.

The remaining eight conditions were dynamic variations of the static configurations described above. For all dynamic conditions there was a single circle that continuously fluctuated in size between the standard and the target. By adjusting the target size, participants adjusted the growth rate of the circle until they perceived it as not changing in size.

##### Stationary-dynamic control

For the Stationary-Dynamic Control condition (Figure 2D), a single circle was presented in isolation. The circle smoothly changed in size from the standard (1.8 or 2.0°) to the (adjustable) target size and then back to the standard size. The participant manipulated the growth rate of the circle by directly adjusting the size of the circle when it was in the target position, within a range of extremes represented by a target that shrank to 0.2° or grew to 5.75° (matching the static conditions). A full animation cycle (from the standard position to the target position and back to the standard position) took 1.4 s and was repeated for as long as the participant needed to complete the trial.

##### Stationary-dynamic Ebbinghaus

The Stationary-Dynamic Ebbinghaus condition (Figure 2E) was the same as the Stationary-Dynamic Control condition, with the addition of dynamic inducers. Small inducers always surrounded the standard and large inducers always surrounded the (adjustable) target. The inducers smoothly fluctuated in size and position from small (0.7° width, 1.5°eccentricity) to large (3.75° width, 4.75° eccentricity) across the animation. The duration of the animation cycle and the size of the standard and target were the same as for the other stationary target-dynamic context conditions.

##### Stationary-dynamic corridor

The Stationary-Dynamic Corridor condition (Figure 2F) was the same as the Stationary-Dynamic Control condition, with the addition of the grid floor background. For this condition, the standard and target were stationary, and did not change position throughout the trial. However, the grid floor translated vertically such that the standard appeared at the top of the corridor and the target appeared at the bottom of the corridor. The duration of the animation cycle and the size of the standard and target were matched to the other stationary target-dynamic context conditions.

The remaining conditions added diagonal or vertical translation of the target, in addition to the same context dynamics described for the above conditions. The Moving-Dynamic conditions of Experiment 1 (Figure 2, third row) included a moving target surrounded by a moving context. These conditions test for an interaction between target and context dynamics, as predicted by our hypothesis.

##### Moving-dynamic control

The Moving-Dynamic Control condition (Figure 2G) involved a single circle translating diagonally from the upper left (−3.5° left and 4° up; the standard) to the bottom right (3.5° right and −3° down; the target) quadrants of the screen, using the same standard and target positions as the Static conditions. The translation path was on a 45° angle spanning 9.9°. A full animation loop took 1.4 s to complete fully (standard to target and back to standard). Thus, the circle moved at a rate of 14.1°/s.

##### Moving-dynamic Ebbinghaus

The Moving-Dynamic Ebbinghaus condition (Figure 2H) was similar to the Stationary-Dynamic Ebbinghaus with the added diagonal translation of the entire stimulus. It was also the same as the Moving-Dynamic Control condition, with the addition of dynamic inducers (small and near for the standard position, large and far for the target position). The position of the standard and target, the sizes of the standard and target, and the translation path of the circle matched the other moving target-moving context conditions.

##### Moving-dynamic corridor

The Moving-Dynamic Corridor condition (Figure 2I) was the same as the Stationary-Dynamic Control condition, with the addition of a horizontally translating (7° extent) grid floor background. This resulted in a target moving obliquely so that its relative motion was vertical along a corridor background that itself moved horizontally. The standard and target positions and sizes and the translation path of the circle matched the moving target-moving context conditions. The position of the standard and target, the sizes of the standard and target, and the translation path of the circle matched the moving target-moving context conditions.

The final two moving target-stationary context conditions of Experiment 1 (Figure 2, bottom row) included target motion on a stationary background context. This allowed us to test the effects of context motion on illusion magnitudes, in the absence of target motion. We excluded a corresponding Ebbinghaus condition with a translating target and a stationary context as the target would overlap with the surrounding inducers and violate the basic arrangement of the illusion.

##### Moving-stationary control

The Moving-Stationary Control condition (Figure 2J) was similar to the Moving-Dynamic Control condition, except that the circle translated vertically from the standard position (4° up) to the target position (−3° down). The translation path spanned 7°, leading to a speed of 10°/s over the full 1.4 s animation cycle. The sizes of the standard and target matched the other conditions.

##### Moving-stationary corridor

The Moving-Stationary Corridor condition (Figure 2K) was similar to the Moving-Stationary Control condition with the addition of a static grid floor background. The position of the standard and target, the sizes of the standard and target, and the translation path of the circle matched the Moving-Stationary Control condition. This condition included target translation without any movement or change in the background context. In this way, it is similar to the Dynamic Corridor illusion described in Mruczek et al. (2020).

#### Quantifying point of subjective equality and illusion magnitudes

The response on each trial is a measure of the participant’s PSE, which for our purpose is the physical size of the target circle such that the participant perceived that the target was not changing size over time (dynamic trials) or such that participant perceived that the target and standard were the same size (static trials). To collapse across the two different standard sizes (1.8 and 2.0°), PSEs are reported as a percentage of the standard size. Positive PSE values indicate that the target circle was set by the participant to be physically larger than the standard circle. We used a conservative approach to limit the influence of potential outlier trials. Separately for each participant and condition, the trials with the highest and lowest recorded PSEs were discarded prior to averaging.

Perceived size is affected by a variety of factors beyond the specific variables of interest in the current study. For example, we previously reported that for similar stimuli, circles that are lower on the screen are perceived to be slightly smaller (Mruczek et al., 2015), likely due to the fact that objects lower in the visual field tend to be closer in a 3D world (Roelofs and Zeeman, 1957; Sonoda, 1961; Dunn et al., 1965). To isolate the influence of motion dynamics and context, we calculated the difference between PSEs for trials with an Ebbinghaus or Corridor context and the corresponding Control condition containing matched position and circle trajectory parameters (same row of Figure 2). This measure, which we refer to as the illusion magnitude, was calculated for the seven conditions that included an Ebbinghaus or Corridor context (middle and right columns of Figure 2). The illusion magnitude also minimizes the influence of any potential response bias in our participants. If there was no effect of the surrounding context or motion dynamics on the perceived size of the circles, then we would expect illusion magnitudes to be zero. As with PSEs, positive illusion magnitudes indicate that the target circle was set by the participant to be physically larger than the standard circle. For dynamic trials, this corresponds to a circle that physically changed size over the animation period.

#### Statistical analysis

Statistical analyses for all experiments were performed in MATLAB and IMB SPSS Statistics 28 (IBM, Armonk, NY, United States; RRID:SCR_016479). To compare illusion magnitudes across conditions, we used a series of standard parametric tests. To determine if each condition led to a significant illusory effect, we compared illusion magnitudes for each condition against zero using a one-sample *t*-test. Pairwise comparisons across conditions were performed using paired *t*-tests. These paired comparisons followed an initial repeated-measures ANOVA to verify a main effect of condition. For all statistical tests, we used an α of 0.05 with a conservative Bonferroni correction. Given our within-subjects design, we calculated confidence intervals with between-subject variance removed (Cousineau, 2005; Morey, 2008). We note that the statistical comparisons presented in all tables were qualitatively similar when using Wilcoxon sign-rank tests or non-parametric permutation tests (and a conservative Bonferroni correction) and did not change interpretation of the reported results.

### Results and discussion

**FIGURE 3:**
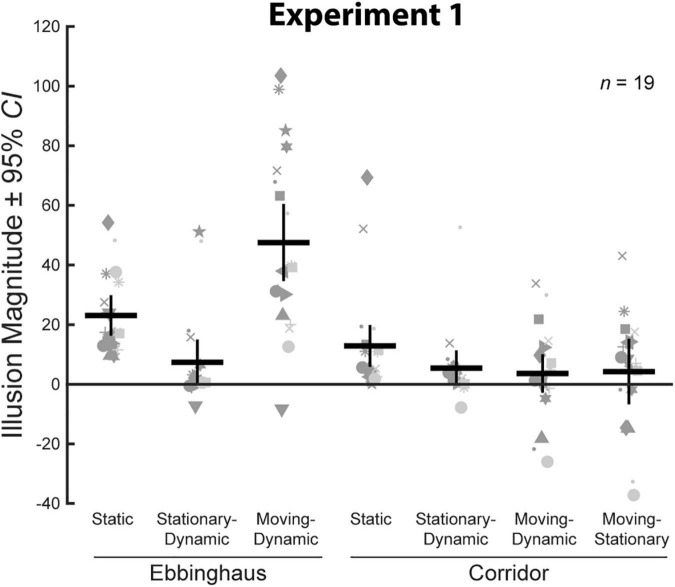
Illusion magnitudes for the seven conditions with a surrounding context in Experiment 1. Gray markers indicate individual participant data. Black horizonal lines indicate group means and black vertical lines represent 95% CIs with between-subject variance removed. Statistical comparisons against zero and across conditions are summarized in Table 1.

We compared average illusion magnitudes for the seven conditions with an Ebbinghaus or Corridor context for Experiment 1. If there were no illusory effects, we would expect illusion magnitudes to be zero. Means, confidence intervals, one-sample *t*-tests against zero for all conditions, and paired *t*-tests for all pairwise comparisons are summarized in Table 1. Individual and group-averaged illusion magnitudes are shown in Figure 3. Importantly, both the Static Ebbinghaus and the Static Corridor conditions showed a significant illusory effect. This demonstrates that our stimulus parameters (size, spacing, etc.) led to consistent illusory percepts for the classic illusion configurations, even for the simplified corridor with the grid floor.

**TABLE 1:** One-sample and pairwise paired *t*-tests for all conditions with surrounding context of Experiment 1.

	Descriptive statistics	One-sample *t*-test (H_0_: μ = 0)	Paired *t*-tests (H_0_: μ_diff_ = 0)					
	*M* [95% CI]	*t*(18) *p* *d*	*t*(18) *p* *d*					
Condition			Stationary-Dynamic Ebbinghaus	Moving-Dynamic Ebbinghaus	Static Corridor	Stationary-Dynamic Corridor	Moving-Dynamic Corridor	Moving-Stationary Corridor
Static Ebbinghaus	23.1 [16.7, 29.5]	**7.57** **<0.0001** **1.74**	**−3.67** **0.002** **−0.84**	**3.66** **0.002** **0.84**	−3.14 0.006 −0.72	**−5.73** **<0.0001** **−1.32**	**−4.66** **0.0002** **−1.07**	−3.02 0.007 −0.69
Stationary-Dynamic Ebbinghaus	7.4 [−0.3, 15.1]	2.02 0.058 0.46		**6.34** **<0.0001** **1.45**	1.04 0.31 0.24	−0.72 0.48 −0.17	−0.84 0.41 −0.19	−0.49 0.63 −0.11
Moving-Dynamic Ebbinghaus	47.5 [32.7, 62.4]	**6.72** **<0.0001** **1.54**			**−5.90** **<0.0001** **−1.35**	**−6.05** **<0.0001** **−1.39**	**−6.15** **<0.0001** **−1.41**	**−5.51** **<0.0001** **−1.26**
Static Corridor	12.9 [4.2, 21.5]	**3.12** **0.006** **0.72**				−1.72 0.10 −0.39	−2.22 0.04 −0.51	−1.47 0.16 −0.34
Stationary-Dynamic Corridor	5.4 [−0.4, 11.3]	1.94 0.068 0.45					−0.58 0.57 −0.13	−0.21 0.84 −0.05
Moving-Dynamic Corridor	3.6 [−3.8, 11.1]	1.02 0.32 0.23						0.15 0.88 0.04
Moving-Stationary Corridor	4.3 [−4.9, 13.4]	0.98 0.34 0.23						

Bold cells denote significant effects (α_Bonferonni_ = 0.007 for 7 one-sample tests; α_Bonferonni_ = 0.002 for 21 pairwise comparisons).

We verified that illusion magnitudes differed across conditions using a repeated measures ANOVA. Mauchly’s test revealed a violation of sphericity, ε = 0.56, χ^2^(20) = 52.1, *p* < 0.001, and so we report the Greenhouse–Geisser correction. There was a significant main effect of condition, *F*(3.37,60.71) = 17.73, *p* < 0.001, $\eta_{p}^{2}$ = 0.50. Below, we focus on specific comparisons related to our *a priori* hypotheses using Bonferroni-corrected *post-hoc* tests.

First, we compared illusion magnitudes across the three Ebbinghaus configurations as a verification of our previous results (Mruczek et al., 2015, 2020). The results of these comparisons were in complete agreement with our previous work. The Moving-Dynamic Ebbinghaus, in which the target and inducers translated, led to a significantly larger illusory effect than the Static Ebbinghaus. In contrast, the Stationary-Dynamic Ebbinghaus, in which the target was stationary, led to a significantly weaker illusory effect than the Static Ebbinghaus. Finally, the Moving-Dynamic Ebbinghaus led to a significantly stronger effect than the Stationary-Dynamic Ebbinghaus. Thus, for the Ebbinghaus configuration, a combination of target and context motion led to the largest effect for the Ebbinghaus configurations.

In contrast, no pairwise comparisons between Corridor conditions reached significance (Table 1). Consistent with our previous results (Mruczek et al., 2020), the Moving-Stationary Corridor, in which a translating target moved along a stationary background, led to a weaker illusion than the Static Corridor, although this was not statistically significant when correcting for multiple comparisons. Importantly, the Diagonally Moving-Dynamic Corridor, which added a translational component to the background, also led to a weaker illusion (though not significantly) compared to the Static Corridor. Thus, the translation of the contextual background, whether in conjunction with a moving target or not, did not enhance the magnitude of the Corridor illusion relative to the classic configuration. If anything, these manipulations reduced the magnitude of the illusion in a manner similar to that observed with a target moving across a stationary background.

## Experiment 2: Does a dynamically changing context enhance the corridor illusion?

In Experiment 1, we found that the combination of target and context motion led to an enhanced illusory effect for the Ebbinghaus illusion, but not for the Corridor illusion. One additional difference between these two dynamic illusions is that the context itself changes for dynamic versions of the Ebbinghaus illusion. For example, inducers grow and shrink (in addition to translating) in the Moving-Dynamic Ebbinghaus conditions of Experiment 1. In contrast, the grid floor translates but does not dynamically change in the Moving-Dynamic Corridor condition of Experiment 1. In Experiment 2, we tested whether the combination of target motion and a dynamically changing context was necessary for producing particularly large illusion magnitudes for dynamic size illusions.

### Materials and methods

#### Participants

Experiment 2 included 15 participants (five female), including three authors. Five participants also participated in Experiment 1. All participants reported normal or corrected-to-normal vision and all participants, except the authors, were naïve to the specific aims and designs of the experiments. Each participant signed an informed consent form before participating and was paid $8 upon completion of the experiment. All procedures were approved by the College of the Holy Cross Institutional Review Board before conducting any of the experiments.

Using a conservative estimated effect size of *d* = 0.80 based on our previous studies, a two-tailed test at α = 0.05 and a related-samples design with a sample size of 15 yields an expected power (1–β) of 0.82 for Experiment 2.

#### Display

Experiment 2 was performed on the same experimental setup as Experiment 1.

#### Experimental design and conditions

**FIGURE 4:**
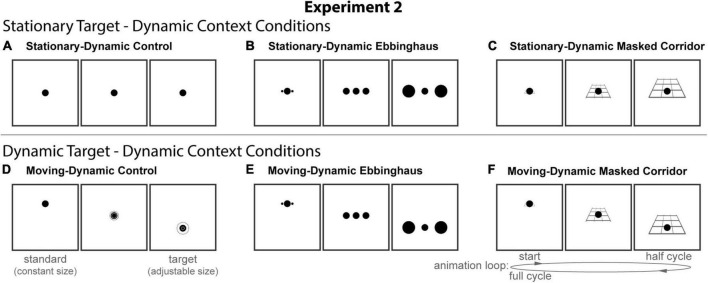
The six conditions of Experiment 2. Columns represent the three distinct contexts [Control **(A,D)**, Ebbinghaus **(B,E)**, Masked Corridor **(C,F)**]. Rows represent the two configurations [Stationary-Dynamic **(A–C)** and Moving-Dynamic **(D–F)**]. For each condition, screenshots for the beginning (standard circle), middle, and end (target circle) frames are shown. The animation [as labeled in panel **(F)**] played in a continuous loop. In the figure, the circle is the same physical size in all frames; during the experiment, the target (lower right circle for Static conditions or the far-right frame for Dynamic conditions) was adjusted by the participant [see panel **(D)**]. Here, stimuli are cropped to display the central portion of the screen to save space. Supplementary Video 2 showing all of the dynamic conditions for Experiment 2 can be found in the Supplementary materials (best viewed in “loop” mode).

All stimuli were composed of black geometric shapes presented on a white background. Each participant was shown six distinct conditions (Figure 4) formed from crossing three contexts (Control, Ebbinghaus, and Masked Corridor) and two configurations (Stationary-Dynamic and Moving-Dynamic). Supplementary Video 2 showing all dynamic conditions can be found in the Supplementary materials (best viewed in “loop” mode). Each participant completed 12 trials per condition for a total of 72 trials. Participants were allowed to take self-timed breaks after every 20% of completed trials. Experiment 2 used the same method of adjustment task used in all other experiments. As Experiment 2 only included dynamic conditions, there was a single circle that continuously fluctuated in size between the standard and the target. By adjusting the target size, participants adjusted the growth rate of the circle until they perceived it as not changing in size (illustrated in Figure 4D). There were no time limits; participants could take as much time as needed to make their response. Participants were allowed to freely view the display.

The stationary-dynamic conditions of Experiment 2 (Figure 4, top row) included a dynamically changing context surrounding a stationary target. Based on our previous results, we anticipate that illusion magnitudes would be weak for these conditions.

##### Stationary-dynamic control

The Stationary-Dynamic Control condition (Figure 4A) was identical to the same condition in Experiment 1.

##### Stationary-dynamic Ebbinghaus

The Stationary-Dynamic Ebbinghaus condition (Figure 4B) was the same as the Stationary-Dynamic Control condition, with the addition of dynamic inducers. The size and eccentricity of the inducers was the same as for the complementary condition of Experiment 1, except that there were only two inducers flanking the central circle.

##### Stationary-dynamic masked corridor

The Stationary-Dynamic Masked Corridor condition (Figure 4C) was the same as the Stationary-Dynamic Control condition, with the addition of the grid floor background. Additionally, we introduced a modified corridor context in which only a portion of the grid floor was visible at any time in the animation. For the standard, corresponding to the “far” position of the corridor, the vertical extent of the visible grid floor was 0.7°; for the target, corresponding to the “near” position of the corridor, the vertical extent was 3.75°. The full horizontal extent of the grid floor was always visible. Other than the masking of the grid floor, this condition was identical to the Stationary-Dynamic Corridor condition of Experiment 1. However, due to the masking, the grid flood did not appear to translate. Rather, it dynamically changed in size over time.

The Moving-Dynamic conditions of Experiment 2 (Figure 4, bottom row) added vertical translation of the target, in addition to the same types of context dynamics described for the above conditions. We hypothesized that this would lead to an increased illusion magnitude for both the Ebbinghaus and Corridor illusions.

##### Moving-dynamic control

The Moving-Dynamic Control condition (Figure 4D) was identical to the Moving-Stationary Control condition in Experiment 1.

##### Moving-dynamic Ebbinghaus

The Moving-Dynamic Ebbinghaus condition (Figure 4E) was the same as the Moving-Dynamic Control condition, with the addition of two dynamic inducers flanking the central circle.

##### Moving-dynamic masked corridor

The Moving-Dynamic Masked Corridor condition (Figure 4F) was similar to the Stationary-Dynamic Masked Corridor condition with the added vertical translation of the circle and masked grid floor. Other than the masking of the grid floor, this condition was identical to the Moving-Stationary Corridor condition of Experiment 1, with the grid flood appearing to dynamically change in size over time.

#### Quantifying point of subjective equality and illusion magnitudes

Point of subjective equality and illusion magnitudes were computed using an identical procedure to that described for Experiment 1.

#### Statistical analysis

The statistical procedures were identical to those described for Experiment 1.

### Results and discussion

**FIGURE 5:**
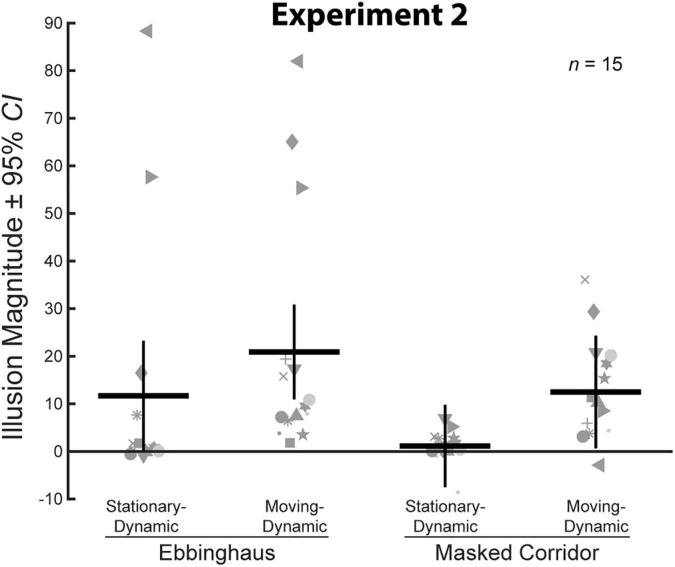
Illusion magnitudes for the four conditions with a surrounding context in Experiment 2. Conventions are the same as in Figure 3. Statistical comparisons against zero and across conditions are summarized in Table 2.

We compared average illusion magnitudes for the four conditions with an Ebbinghaus or Corridor context. If there were no illusory effects, we would expect illusion magnitudes to be zero. Means, confidence intervals, and one-sample *t*-tests against zero for all conditions, and paired *t*-tests for all pairwise comparisons are summarized in Table 2. Individual and group-averaged illusion magnitudes are shown in Figure 5.

**TABLE 2:** One-sample and pairwise paired *t*-tests for all conditions with surrounding context of Experiment 2.

	Descriptive statistics	One-sample *t*-test (H_0_: μ = 0)	Paired *t*-tests (H_0_: μ_diff_ = 0)		
	*M* [95% CI]	*t*(14) *p* *d*	*t*(14) *p* *d*		
Condition			Moving-Dynamic Ebbinghaus	Stationary-Dynamic Masked Corridor	Moving-Dynamic Masked Corridor
Stationary-Dynamic Ebbinghaus	11.7 [−2.7, 26.0]	1.74 0.10 0.45	2.74 0.016 0.71	−1.60 0.13 −0.41	0.10 0.92 0.03
Moving-Dynamic Ebbinghaus	20.9 [7.0, 34.8]	**3.22** **0.006** **0.83**		**−3.09** **0.008** **−0.80**	−1.18 0.26 −0.32
Stationary-Dynamic Masked Corridor	1.2 [−0.7, 3.0]	1.33 0.21 0.34			**4.27** **0.0008** **1.10**
Moving-Dynamic Masked Corridor	12.5 [6.5, 18.5]	**4.47** **0.0005** **1.15**			

Bold cells denote significant effects (α_Bonferonni_ = 0.0125 for 4 one-sample tests; α_Bonferonni_ = 0.008 for six pairwise comparisons).

A two-way repeated measures ANOVA revealed a significant main effect of motion, *F*(1,14) = 13.79, *p* = 0.002, $\eta_{p}^{2}$ = 0.50. On average, illusion magnitudes for the Moving-Dynamic conditions (*M* = 16.7, CI = [9.2, 24.2]) were significantly larger than for the Stationary-Dynamic conditions (*M* = 6.4, CI = [−1.0, 13.8]). There was no main effect of context, *F*(1,14) = 1.97, *p* = 0.18, $\eta_{p}^{2}$ = 0.12, and no interaction between context and motion, *F*(1,14) = 0.72, *p* = 0.41, $\eta_{p}^{2}$ = 0.05. Thus, illusion magnitudes were approximately equated across the Ebbinghaus (*M* = 16.3, CI = [2.6, 30.0]) and Masked Corridor (*M* = 6.8, CI = [3.4, 10.2]) configurations, and the effects of motion were similar for the two illusion types.

As shown in Table 2, the two Moving-Dynamic conditions yielded a significant illusory effect, whereas the two Stationary-Dynamic conditions did not. Pairwise comparisons revealed that the Moving-Dynamic Ebbinghaus resulted in a larger illusion compared to the Stationary-Dynamic Ebbinghaus. However, this comparison was only marginally significant and did not survive correction for multiple comparisons (Table 2). This may be due to the presence of outliers for the Ebbinghaus conditions for some participants (Figure 5). Indeed, when considering median illusion magnitudes, both Stationary-Dynamic conditions did not yield illusory effects (Ebbinghaus *Mdn* = 0.83, Masked Corridor *Mdn* = 0.99), whereas both Moving-Dynamic conditions led to robust illusory effects (Ebbinghaus *Mdn* = 9.56, Masked Corridor Mdn = 10.27). We emphasize that we have replicated the observation that the Moving-Dynamic Ebbinghaus yields much stronger illusion magnitudes compared to the Stationary-Dynamic Ebbinghaus across multiple previous studies (Mruczek et al., 2015, 2020), as well as in Experiment 1.

Importantly, we observed the same pattern of results for the Masked Corridor conditions. The Moving-Dynamic Masked Corridor produced a significantly greater illusion compared to the Stationary-Dynamic Masked Corridor (Table 2 and Figure 5). We emphasize that this is different from what we observed in Experiment 1, when the background grid floor was static and unchanging. In Experiment 2, with a dynamically changing grid floor due to the masking, we observed the same illusion enhancement with target translation that we have consistently reported for the Moving-Dynamic Ebbinghaus. However, we cannot say whether this enhancement led to an illusion magnitude stronger than the classic, Static Corridor illusion, as Experiment 2 did not include a matched static condition.

## Experiment 3: Does a dynamically changing context enhance the Ponzo illusion?

The results from Experiment 2 suggest that the combination of a moving target and a dynamically changing context underlies the enhanced magnitude of dynamic size illusions. Although the results hint at this effect being relevant for both size contrast (e.g., the Ebbinghaus illusion) and size constancy (e.g., the Corridor illusion) illusions, the masking procedure used for the Masked Corridor illusions of Experiment 2 may have introduced size contrast-like effects. For example, a comparison of the endpoints of the animation for the Dynamic Masked Corridor conditions of Experiment 2 (Figures 4C,F) show that the extent of the context and the size of the contextual elements (e.g., grid floor tiles) was larger in the “near” position compared to the “far” position. As such, the masked corridor configurations in Experiment 2 mimic in some ways the size-contrast elements that define the Ebbinghaus Illusion. In Experiment 3, we more directly tested the effects of motion dynamics on size constancy illusions by using full and masked versions of the Ponzo illusion, which contains simpler contextual elements compared to the Corridor illusion.

### Materials and methods

#### Participants

Experiment 3 included 22 participants (seven female), including two authors. All participants reported normal or corrected-to-normal vision and all participants, except the authors, were naïve to the specific aims and designs of the experiments. Each participant signed an informed consent form before participating and was paid $10 upon completion of the experiment. All procedures were approved by the College of the Holy Cross Institutional Review Board before conducting any of the experiments.

Using a conservative estimated effect size of *d* = 0.80 based on our previous studies, a two-tailed test at α = 0.05 and a related-samples design with a sample size of 22 yields an expected power (1–β) of 0.95 for Experiment 3.

#### Display

Data for Experiment 3 was collected remotely due to COVID-19 restrictions, with each participant utilizing their own laptop or desktop computer. Participants received an email with the necessary scripts and instructions for controlling the experiment and a set of practice trials. All stimuli were generated and presented using the Psychophysics Toolbox (Brainard, 1997; Pelli, 1997; Kleiner et al., 2007) for MATLAB. However, there was necessarily some variability in the exact setup for each participant. Participants viewed the stimuli binocularly and without restraint. Mean viewing distance across participants was 47 cm (range = 25–71 cm). The average setup had a mean pixels per degree of visual angle of 41 (range = 22–56). All participants except one (46 Hz) viewed the stimulus on a monitor with an effective frame rate of 60 Hz. Given the robust effects that we have observed in this line of research, we were confident that the added variability from setup differences would not obscure differences across conditions.

#### Experimental design and conditions

**FIGURE 6:**
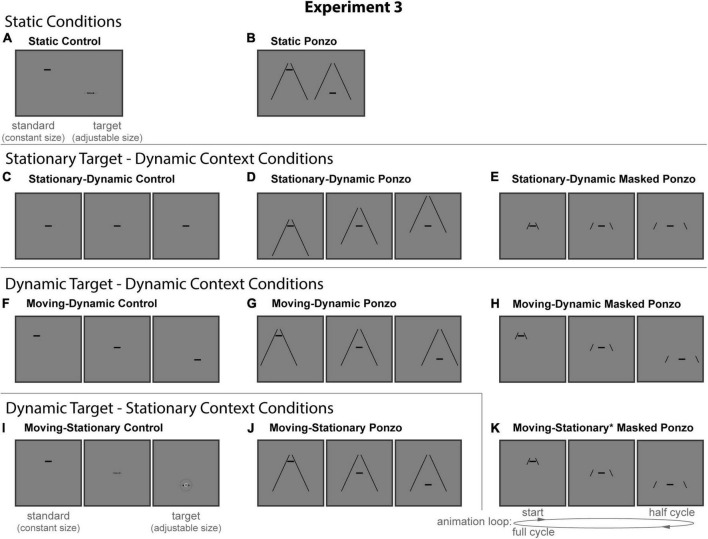
The 11 conditions of Experiment 3. Columns represent the three distinct contexts [Control **(A,C,F,I)**, Ponzo **(B,D,G,J)**, Masked Ponzo **(E,H,K)**]. Rows represent the four configurations [Static **(A,B)**, Stationary-Dynamic **(C–E)**, Moving-Dynamic **(F–H)**, and Moving-Stationary **(I–K)**]. For each condition, screenshots for the beginning (standard line), middle, and end (target line) frames are shown. The animation [as labeled in panel **(K)**] played in a continuous loop. In the figure, the central line is the same physical size in all frames; during the experiment, the target (lower right line for Static conditions or the far-right frame for Dynamic conditions) was adjusted by the participant [see panels **(A,I)**]. Here, stimuli are cropped to display the central portion of the screen to save space. Supplementary Video 3 showing all of the dynamic conditions for Experiment 3 can be found in the Supplementary materials (best viewed in “loop” mode).

All stimuli were composed of black geometric shapes presented on a mid-gray background. Each participant was shown 11 distinct conditions (Figure 6) formed from crossing three contexts (Control, Ponzo, and Masked Ponzo) and four configurations (Static, Stationary-Dynamic, Moving-Dynamic, and Moving-Stationary). Supplementary Video 3 showing all dynamic conditions can be found in the Supplementary materials (best viewed in “loop” mode). Each participant completed 8 trials per condition for a total of 88 trials. Participants were allowed to take self-timed breaks after every 20% of completed trials. Experiment 3 used the same method of adjustment task used in all other experiments. For the static conditions, participants moved a computer mouse up and down to adjust the size of a target line to match the size of a nearby standard line (illustrated in Figure 6A). In the dynamic conditions, participants adjusted the growth rate of a target line until they perceived it as not changing in size over time (illustrated in Figure 6I). There were no time limits; participants could take as much time as needed to make their response. Participants were allowed to freely view the display.

The static conditions of Experiment 3 (Figure 6, top row) replicate the classic Ponzo configurations along with a matched no-context control condition.

##### Static control

The static control condition (Figure 6A) was identical to the same condition in Experiment 1, except that the standard and target were horizontal lines rather than circles. The position, size (i.e., length) of the standard, and range of adjustable target sizes was the same as in Experiment 1.

##### Static Ponzo

The Static Ponzo condition (Figure 6B) was the familiar Ponzo configuration, in which the standard and target were surrounded by two oblique lines. The surrounding context was similar in size and extent to the corridor grid floor of Experiment 1. The surrounding lines spanned a height of 9.6° and were separated by a width of 11° at their base and approximately 1.07° at their apex. The standard (1.8 or 2.0° length) was positioned in the upper portion of the left configuration; the target (adjustable within the range of 0.2–5.75° length) was positioned in the lower portion of the right configuration.

The stationary-dynamic conditions of Experiment 3 (Figure 6, second row) included a dynamically changing context surrounding a stationary target. Based on our previous results, we anticipate that illusion magnitudes would be weak for these conditions.

##### Stationary-dynamic control

The Stationary-Dynamic Control condition (Figure 6C) was similar to the same condition in Experiment 1, except that the standard and target were horizontal lines rather than circles. The line smoothly changed in length from the standard (1.8 or 2.0°) to the (adjustable) target length and then back to the standard length. The participant manipulated the growth rate of the line by directly adjusting the length of the line when it was in the target position, within a range of extremes represented by a target that shrank to 0.2° or grew to 5.75° (matching the Static conditions). A full animation cycle took 1.4 s to complete and was repeated for as long as the participant needed to complete the trial.

##### Stationary-dynamic Ponzo

The Stationary-Dynamic Ponzo condition (Figure 6D) was the same as the Stationary-Dynamic Control condition, with the addition of the surrounding Ponzo context. For this condition, the horizonal line was stationary, and did not change position throughout the trial. Instead, the contextual lines translated vertically such that the standard appeared at the top of the configuration and the target appeared at the bottom of the configuration. Thus, the target line was stationary and surrounded by a translating context. The duration of the animation cycle and the size of the standard and target were matched to the other stationary target-dynamic context conditions.

##### Stationary-dynamic masked Ponzo

The Stationary-Dynamic Masked Ponzo condition (Figure 6E) was the same as the Stationary-Dynamic Ponzo condition, but only a portion of the surrounding contextual lines was visible at any time in the animation. This “masked” Ponzo configuration was similar to the Masked Corridor conditions of Experiment 2. However, the vertical extent of the mask was always 1.0°, avoiding the potentially confounding change in the size and extent of the contextual elements surrounding the standard and target positions. Due to the masking, the contextual surround did not appear to translate vertically, but instead appeared to translate away and toward the central line over the animation cycle.

The remaining conditions added diagonal or vertical translation of the target, in addition to the same context dynamics described for the above conditions. The Moving-Dynamic conditions of Experiment 3 (Figure 6, third row) included a moving target surrounded by a moving context. These conditions test for an interaction between target and context dynamics, as predicted by our hypothesis.

##### Moving-dynamic control

The Moving-Dynamic Control condition (Figure 6F) was the same as the corresponding condition in Experiment 1, but used a central line rather than a circle. The line translated diagonally from the upper left (−3.5° left and 4° up; the standard) to the bottom right (3.5° right and −3° down; the target) quadrants of the screen, using the same standard and target positions as the Static conditions. The translation path was on a 45° angle spanning 9.9°. A full animation loop took 1.4 s to complete fully (standard to target and back to standard). Thus, the line moved at a rate of 14.1°/s.

##### Moving-dynamic Ponzo

The Moving-Dynamic Ponzo condition (Figure 6G) was similar to the Stationary-Dynamic Ponzo condition with the added diagonal translation of the central line and horizontal translation (7° extent) of the Ponzo line context. The position of the standard and target, the lengths of the standard and target, and the translation path of the central line matched the other moving target-dynamic context conditions.

##### Moving-dynamic masked Ponzo

The Moving-Dynamic Masked Ponzo condition (Figure 6H) was similar to the Stationary-Dynamic Masked Ponzo condition with the added diagonal translation of the central line and horizontal translation (7° extent) of the masked Ponzo line context. The position of the standard and target, the lengths of the standard and target, and the translation path of the central line matched the other moving target-dynamic context conditions. Compared to the Moving-Dynamic Ponzo, this condition included a perceived change in the eccentricity of the surrounding context, in addition to the global translation of the stimulus elements.

The final three moving target-stationary context conditions of Experiment 3 (Figure 6, bottom row) included vertical target motion on a stationary background context. It should be noted that the context was not strictly stationary for the Moving-Stationary* Masked Ponzo condition (described below), because of the apparent change in the eccentricity of the surrounding lines due to the masking procedure. However, the vertical target motion matched that of the other moving target-stationary context conditions, and so we maintain this nomenclature while indicating a strict departure from its description by the asterisk.

##### Moving-stationary control

The Moving-Stationary Control condition (Figure 6I) was similar to the Moving-Dynamic Control condition, except that the target line translated vertically from the standard position (4° up) to the target position (−3° down). The translation path spanned 7°, leading to a speed of 10°/s over the full 1.4 s animation cycle. The sizes of the standard and target matched the other conditions.

##### Moving-stationary Ponzo

The Moving-Stationary Corridor condition (Figure 6J) was similar to the Moving-Stationary Control condition with the addition of a static Ponzo lines background. The position of the standard and target, the lengths of the standard and target, and the translation path of the target line matched the Moving-Stationary Control condition. Similar to the Moving-Stationary Corridor condition of Experiment 1, this condition included target translation without any movement or change in the background context.

##### Moving-stationary* masked Ponzo

The Moving-Stationary* Masked Ponzo condition (Figure 6K) was similar to the Moving-Stationary Corridor condition, but only a portion of the surrounding contextual lines was visible at any time in the animation. The position of the standard and target, the lengths of the standard and target, and the translation path of the central line matched the other Moving-Stationary contexts. As noted above, unlike the Moving-Stationary Ponzo, this condition included a perceived change in the eccentricity of the surrounding context, in addition to the vertical translation of the stimulus elements. Thus, strictly speaking, this condition was very similar to the Moving-Dynamic Masked Ponzo, but with vertical, instead of oblique, target motion.

#### Quantifying point of subjective equality and illusion magnitudes

Point of subjective equality and illusion magnitudes were computed using an identical procedure to that described for Experiment 1.

#### Statistical analysis

The statistical procedures were identical to those described for Experiment 1.

### Results and discussion

**FIGURE 7:**
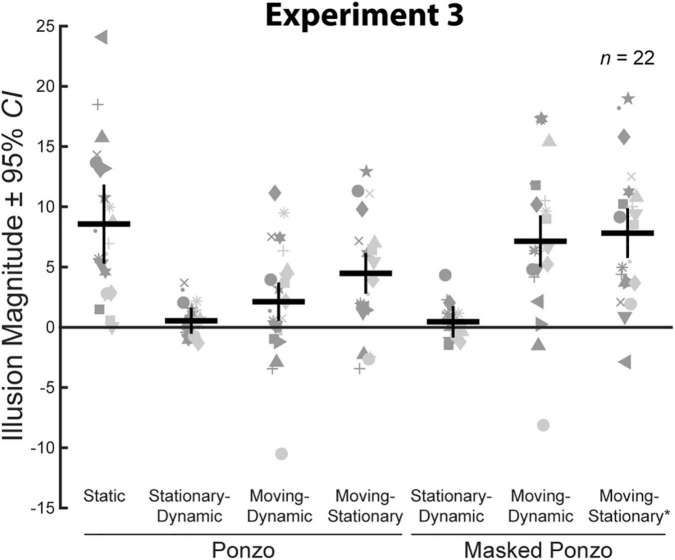
Illusion magnitudes for the seven conditions with a surrounding context in Experiment 3. Conventions are the same as in Figure 3. Statistical comparisons against zero and across conditions are summarized in Table 3.

We compared average illusion magnitudes for the seven conditions with a surrounding context for Experiment 3. If there were no illusory effects, we would expect illusion magnitudes to be zero. Means, confidence intervals, one-sample *t*-tests against zero for all conditions, and paired *t*-tests for all pairwise comparisons are summarized in Table 3. Individual and group-averaged illusion magnitudes are shown in Figure 7. The Static Ponzo condition showed a significant illusory effect demonstrating that our stimulus parameters (size, spacing, etc.) led to consistent illusory percepts for the classic illusion configuration.

**TABLE 3:** One-sample and pairwise paired *t*-tests for all conditions with surrounding context of Experiment 3.

	Descriptive statistics	One-sample *t*-test (H_0_: μ = 0)	Paired *t*-tests (H_0_: μ_diff_ = 0)					
	*M* [95% CI]	*t*(21) *p* *d*	*t*(21) *p* *d*					
Condition			Stationary-Dynamic Ponzo	Moving-Dynamic Ponzo	Moving-Stationary Ponzo	Stationary-Dynamic Masked Ponzo	Moving-Dynamic Masked Ponzo	Moving-Stationary* Masked Ponzo
Static Ponzo	8.6 [5.8, 11.4]	**6.39** **<0.0001** **1.36**	**−6.01** **<0.0001** **−1.28**	**−3.87** **0.0009** **−0.83**	−2.28 0.033 −0.49	**−6.67** **<0.0001** **−1.42**	−0.70 0.49 −0.15	−0.37 0.71 −0.08
Stationary-Dynamic Ponzo	0.5 [−0.05, 1.1]	1.90 0.07 0.40		1.76 0.09 0.37	**4.22** **0.0004** **0.90**	−0.29 0.78 −0.06	**5.67** **<0.0001** **1.21**	**6.69** **<0.0001** **1.43**
Moving-Dynamic Ponzo	2.1 [−0.01, 4.3]	2.07 0.051 0.44			2.41 0.025 0.51	−1.71 0.10 −0.37	**5.35** **<0.0001** **1.14**	**4.89** **0.0001** **1.04**
Moving-Stationary Ponzo	4.5 [2.3, 6.7]	**4.24** **0.0004** **0.90**				**−3.92** **0.0008** **−0.84**	2.44 0.023 0.52	**3.76** **0.001** **0.80**
Stationary-Dynamic Masked Ponzo	0.5 [−0.1, 1.0]	1.67 0.11 0.36					**5.10** **<0.0001** **1.09**	**6.14** **<0.0001** **1.31**
Moving-Dynamic Masked Ponzo	7.1 [4.5, 9.8]	**5.59** **<0.0001** **1.19**						0.72 0.48 0.15
Moving-Stationary* Masked Ponzo	7.8 [5.3, 10.3]	**6.53** **<0.0001** **1.39**						

Bold cells denote significant effects (α_Bonferonni_ = 0.007 for 7 one-sample tests; α_Bonferonni_ = 0.002 for 21 pairwise comparisons).

We verified that illusion magnitudes differed across conditions using a repeated measures ANOVA. Mauchly’s test revealed a violation of sphericity, ε = 0.41, χ^2^(20) = 84.9, *p* < 0.001, and so we report the Greenhouse–Geisser correction. There was a significant main effect of condition, *F*(2.44,51.19) = 15.34, *p* < 0.001, $\eta_{p}^{2}$ = 0.42. Below, we focus on specific comparisons related to our *a priori* hypotheses using Bonferroni-corrected *post-hoc* tests.

First, we compared illusion magnitudes across the three Ponzo configurations, in which the full extent of the surrounding lines was visible throughout the trial. As we have previously observed for the Dynamic Ebbinghaus, the Stationary-Dynamic Ponzo condition led to a very weak illusory effect, significantly weaker than the classic Static Ponzo. Both Moving-Dynamic and Moving-Stationary Ponzo conditions led to slightly stronger illusions than the Stationary-Dynamic Ponzo condition, but this was only significant for the Moving-Stationary Ponzo. Overall, these results are generally consistent with the results from Experiment 1. A moving target with a non-dynamic background, even if the background is translating, is not sufficient for inducing larger illusory effects compared to the classic, static illusion.

Next, we compared illusion magnitudes across the three Masked Ponzo configurations. Importantly, since only a portion of the surrounding lines were visible at any point in the trial, the Masked Ponzo conditions included a dynamically changing context, in terms of the eccentricity of the surrounding elements relative to the central target. As with other illusory configurations, the Stationary-Dynamic Masked Ponzo condition did not induce a significant illusory effect and was significantly weaker than the classic Static Ponzo illusion. In contrast, both Moving-Dynamic Masked Ponzo conditions led to significantly stronger illusions relative to the Stationary-Dynamic Masked Ponzo, approximately equal in magnitude to the Static Ponzo illusion.

As a more direct test of the interactive effects of target motion and a dynamically changing background on illusion magnitudes, we compared the Moving-Dynamic Ponzo (translating context) and Moving-Stationary Ponzo (stationary context) conditions with the Moving-Dynamic Masked Ponzo and Moving-Stationary* Masked Ponzo (dynamic context), respectively. In both cases, the masked Ponzo versions led to a significantly stronger illusory effect (Table 3 and Figure 7). Taken together, these results are consistent with the hypothesis that illusory changes in size are enhanced when two factors are present: (1) a moving target and (2) a dynamically changing context.

## Experiment 4: Improved control for masked Ponzo illusion

In Experiment 3, although the Moving-Dynamic Masked Ponzo conditions led to a significantly stronger illusion than the Stationary-Dynamic Masked Ponzo condition, the illusion magnitude did not exceed that of the classic Static Ponzo. In contrast, we have consistently observed much stronger illusion magnitudes for the Moving-Dynamic Ebbinghaus compared to the classic Static Ebbinghaus (Mruczek et al., 2015, 2020; Experiment 1). One reason for this may be that the masked Ponzo conditions represent a drastic reduction in the contextual elements. In particular, depth cues may be weakened by the lack of the extended surrounding lines. The results from Experiment 3 could not rule this out because it did not include a Static Masked Ponzo condition (empty upper right panel of Figure 6), limiting the inferences that can be drawn from the data. In Experiment 4, we replicate the results for the Dynamic Masked Ponzo variants of Experiment 3 and include the corresponding Static Masked Ponzo condition.

### Materials and methods

#### Participants

Experiment 4 included 26 participants (17 female), including one author, 22 of whom also completed Experiment 5 (see below). All participants reported normal or corrected-to-normal vision and all participants, except the author, were naïve to the specific aims and designs of the experiments. Each participant signed an informed consent form before participating and received course credit through the psychology department PSYC 100 participant pool at the completion of the experiment. All procedures were approved by the College of the Holy Cross Institutional Review Board before conducting any of the experiments.

Using a conservative estimated effect size of *d* = 0.80 based on our previous studies, a two-tailed test at α = 0.05 and a related-samples design with a sample size of 26 yields an expected power (1–β) of 0.97 for Experiment 4.

#### Display

Experiment 5 was performed on the same experimental setup as Experiment 1.

#### Experimental design and conditions

**FIGURE 8:**
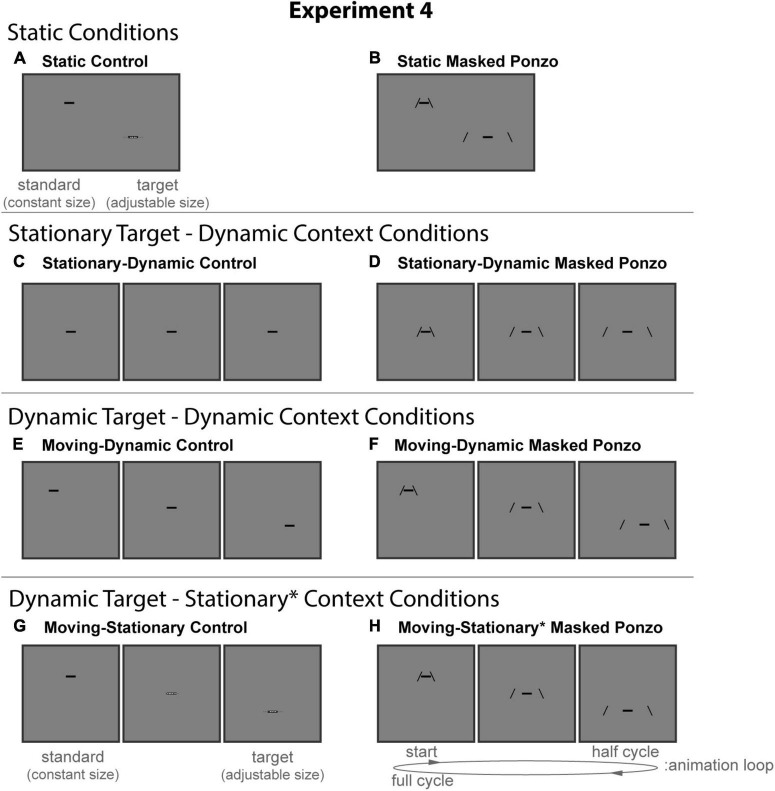
The eight conditions of Experiment 4. Columns represent the two distinct contexts [Control **(A,C,E,G)**, Masked Ponzo **(B,D,F,H)**]. Rows represent the four configurations [Static **(A,B)**, Stationary-Dynamic **(C,D)**, Moving-Dynamic **(E,F)**, Moving-Stationary **(G,H)**]. For each condition, screenshots for the beginning (standard circle), middle, and end (target circle) frames are shown. The animation [as labeled in panel **(H)**] played in a continuous loop. In the figure, the horizontal line is the same physical size in all frames; during the experiment, the target (lower right line for Static conditions or the far-right frame for Dynamic conditions) was adjusted by the participant [see panels **(A,G)**]. Here, stimuli are cropped to display the central portion of the screen to save space. Supplementary Video 4 showing all of the dynamic conditions for Experiment 4 can be found in the Supplementary materials (best viewed in “loop” mode).

All stimuli were composed of black geometric shapes presented on a mid-gray background. Each participant was shown 8 distinct conditions (Figure 8) formed from crossing two contexts (Control, Masked Ponzo) and four configurations (Static, Stationary-Dynamic, Moving-Dynamic, and Moving-Stationary*). Supplementary Video 4 showing all dynamic conditions can be found in the Supplementary materials (best viewed in “loop” mode). Each participant completed 8 trials per condition for a total of 64 trials. Participants were allowed to take self-timed breaks after every 20% of completed trials. Experiment 4 used the same method of adjustment task used in all other experiments. For the Static conditions, participants moved a computer mouse up and down to adjust the size of a target line to match the size of a nearby standard line (illustrated in Figure 8A). In the Dynamic conditions, participants adjusted the growth rate of a target line until they perceived it as not changing in size over time (illustrated in Figure 8G). There were no time limits; participants could take as much time as needed to make their response. Participants were allowed to freely view the display.

The static conditions of Experiment 4 (Figure 8, top row) include a masked variant of the classic Ponzo configurations as a better control for the masked Ponzo illusion than used in Experiment 3. Specifically, the two comparison stimuli matched to the endpoints of the corresponding dynamic masked conditions.

##### Static control

The static control condition (Figure 8A) was identical to the same condition in Experiment 3.

##### Static masked Ponzo

The Static Masked Ponzo condition (Figure 8B) was similar to the Static Ponzo condition (Figure 6B) in Experiment 3, except that the full extent of the surrounding contextual lines was limited to match the endpoints of the animation cycle for the dynamic masked Ponzo conditions. Specifically, the vertical extent of the mask was always 1.0° around the standard (upper left) and target (lower right).

The stationary-dynamic conditions of Experiment 4 (Figure 6, second row) included a dynamically changing context surrounding a stationary target. We anticipate that illusion magnitudes would be weak for these conditions.

##### Stationary-dynamic control

The Stationary-Dynamic Control condition (Figure 8C) was identical to the same condition in Experiment 3.

##### Stationary-dynamic masked Ponzo

The Stationary-Dynamic Masked Ponzo condition (Figure 8D) was identical to the same condition in Experiment 3.

The remaining conditions added diagonal or vertical translation of the target, in addition to the same context dynamics described for the above conditions. The moving target-dynamic context conditions of Experiment 4 (Figure 8, third row) included a moving target surrounded by a context that was both translating (obliquely) and dynamically changing eccentricity.

##### Moving-dynamic control

The Moving-Dynamic Control condition (Figure 8E) was identical to the same condition in Experiment 3.

##### Moving-dynamic masked Ponzo

The Moving-Dynamic Masked Ponzo condition (Figure 8F) was identical to the same condition in Experiment 3.

The final two moving target-stationary context conditions of Experiment 4 (Figure 8, bottom row) included vertical target motion. As with the same conditions in Experiment 5, the context dynamically changed in terms of the eccentricity of the surrounding lines due to the masking procedure. Thus, while we maintain the same naming conventions as in other experiments, we denote this departure from a stationary context with an asterisk.

##### Moving-stationary control

The Moving-Stationary Control condition (Figure 8G) was identical to the same condition in Experiment 3.

##### Moving-stationary* masked Ponzo

The Moving-Stationary* Masked Ponzo condition (Figure 8H) was identical to the same condition in Experiment 3.

#### Quantifying point of subjective equality and illusion magnitudes

Point of subjective equality and illusion magnitudes were computed using an identical procedure to that described for Experiment 1.

#### Statistical analysis

The statistical procedures were identical to those described for Experiment 1.

### Results and discussion

**FIGURE 9:**
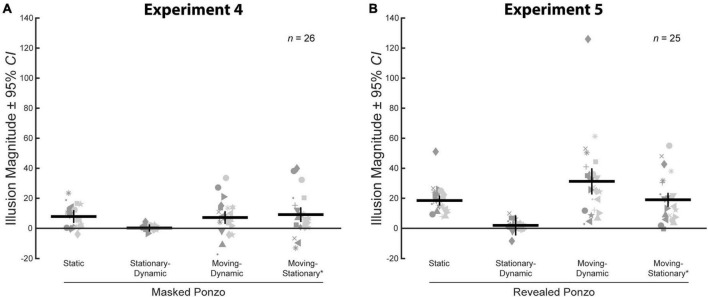
**(A)** Illusion magnitudes for the four conditions with a surrounding context in Experiment 4. Statistical comparisons against zero and across conditions are summarized in Table 4. **(B)** Illusion magnitudes for the four conditions with a surrounding context in Experiment 5. Statistical comparisons against zero and across conditions are summarized in Table 5. Conventions are the same as in Figure 3.

We compared average illusion magnitudes for the four conditions with a surrounding context for Experiment 4. If there were no illusory effects, we would expect illusion magnitudes to be zero. Means, confidence intervals, one-sample *t*-tests against zero for all conditions, and paired *t*-tests for all pairwise comparisons are summarized in Table 4. Individual and group-averaged illusion magnitudes are shown in Figure 9A.

**TABLE 4:** One-sample and pairwise paired *t*-tests for all conditions with surrounding context of Experiment 4.

	Descriptive statistics	One-sample *t*-test (H_0_: μ = 0)	Paired *t*-tests (H_0_: μ_diff_ = 0)		
	*M* [95% CI]	*t*(25) *p* *d*	*t*(25) *p* *d*		
Condition			Stationary-Dynamic Masked Ponzo	Moving-Dynamic Masked Ponzo	Moving-Stationary* Masked Ponzo
Static Masked Ponzo	8.0 [5.2, 10.8]	**5.85** **<0.0001** **1.15**	**−5.71** **<0.0001** **−1.12**	−0.24 0.81 0.05	0.42 0.66 0.08
Stationary-Dynamic Masked Ponzo	0.4 [−0.2, 0.9]	1.31 0.20 0.26		**3.42** **0.002** **0.67**	**3.70** **0.001** **0.73**
Moving-Dynamic Masked Ponzo	7.3 [3.1, 11.6]	**3.53** **0.002** **0.69**			0.74 0.46 0.15
Moving-Stationary* Masked Ponzo	9.3 [4.1, 14.4]	**3.71** **0.001** **0.73**			

Bold cells denote significant effects (α_Bonferonni_ = 0.0125 for 4 one-sample tests; α_Bonferonni_ = 0.008 for 6 pairwise comparisons).

**TABLE 5:** One-sample and pairwise paired *t*-tests for all conditions with surrounding context of Experiment 5.

	Descriptive statistics	One-sample *t*-test (H_0_: μ = 0)	Paired *t*-tests (H_0_: μ_diff_ = 0)		
	*M* [95% CI]	*t*(16) *p* *d*	*t*(16) *p* *d*		
Condition			Stationary-Dynamic Revealed Ponzo	Moving-Dynamic Revealed Ponzo	Moving-Stationary* Revealed Ponzo
Static Revealed Ponzo	18.5 [14.8, 22.2]	**10.3** **<0.0001** **2.07**	**−8.23** **<0.0001** **−1.65**	**3.25** **0.003** **0.65**	0.18 0.86 0.04
Stationary-Dynamic Revealed Ponzo	2.0 [0.5, 3.4]	**2.77** **0.01** **0.55**		**5.42** **<0.0001** **1.08**	**5.56** **<0.0001** **1.11**
Moving-Dynamic Revealed Ponzo	31.2 [20.9, 41.7]	**6.20** **<0.0001** **1.24**			**−3.10** **0.005** **0.62**
Moving-Stationary* Revealed Ponzo	19.0 [12.9, 25.1]	**19.0** **<0.0001** **1.28**			

Bold cells denote significant effects (α_Bonferonni_ = 0.0125 for 4 one-sample tests; *α*_Bonferonni_ = 0.008 for 6 pairwise comparisons).

We verified that illusion magnitudes differed across conditions using a repeated measures ANOVA. Mauchly’s test revealed a violation of sphericity, ε = 0.72, χ^2^(5) = 24.1, *p* < 0.001, and so we report the Greenhouse–Geisser correction. There was a significant main effect of condition, *F*(2.16,53.93) = 5.54, *p* = 0.005, $\eta_{p}^{2}$ = 0.18. Below, we focus on specific comparisons related to our *a priori* hypotheses using Bonferroni-corrected *post-hoc* tests.

The pattern of results for the Masked Ponzo conditions of Experiment 4 is consistent with what was observed in Experiment 3. The classic Static Masked Ponzo condition showed a significant illusory effect of approximately the same magnitude (8.0%) as observed for the Static Ponzo condition of Experiment 3 (8.6%). The Stationary-Dynamic Masked Ponzo condition (stationary target) led to a very weak illusory effect, weaker than the Static Masked Ponzo and not significantly different than zero. Both the Moving-Dynamic Masked Ponzo and Moving-Stationary* Masked Ponzo conditions, in which the target translated and the surrounding elements changed their relative eccentricity, led to a significantly stronger illusion than the Stationary-Dynamic Masked Ponzo, in which the target did not change position. Overall, the results for Experiment 4 were consistent with those from Experiment 3.

## Experiment 5: Enhancing illusion magnitudes in a dynamic Ponzo illusion

In Experiments 4, although the Moving-Dynamic Masked Ponzo conditions led to a significantly stronger illusion than the Stationary-Dynamic Masked Ponzo condition, the illusion magnitude did not exceed that of a matched Static Masked Ponzo. Overall, the results thus far for the size constancy Corridor and Ponzo illusions are still not qualitatively the same as for the size contrast Ebbinghaus, for which the combination of target motion and a dynamic context greatly enhance the illusory effect beyond the classic illusion variant (Mruczek et al., 2015, 2020; Experiment 1). Experiment 4 demonstrated that positional changes in the eccentricity of the surrounding context, while restoring the illusion, did not suffice to enhance illusion magnitudes for the masked Ponzo variants. One reason for this may be that the masked Ponzo conditions represent a drastic reduction in the contextual elements. In particular, depth cues may be weakened by the lack of the extended surrounding lines. In Experiment 5, we present a variation of the dynamic Ponzo illusion that includes (1) target motion, (2) a dynamically changing context, and (3) a more effective surround context leading to a pattern of illusion magnitudes that matches those observed for the Dynamic Ebbinghaus illusion.

### Materials and methods

#### Participants

Experiment 5 included 25 participants (18 female), including one author, 22 of whom also completed Experiment 4 (see above). All participants reported normal or corrected-to-normal vision and all participants, except the author, were naïve to the specific aims and designs of the experiments. Each participant signed an informed consent form before participating and received course credit through the psychology department PSYC 100 participant pool at the completion of the experiment. All procedures were approved by the College of the Holy Cross Institutional Review Board before conducting any of the experiments.

Using a conservative estimated effect size of *d* = 0.80 based on our previous studies, a two-tailed test at α = 0.05 and a related-samples design with a sample size of 25 yields an expected power (1–β) of 0.97 for Experiment 5.

#### Display

Experiment 5 was performed on the same experimental setup as Experiments 1.

#### Experimental design and conditions

**FIGURE 10:**
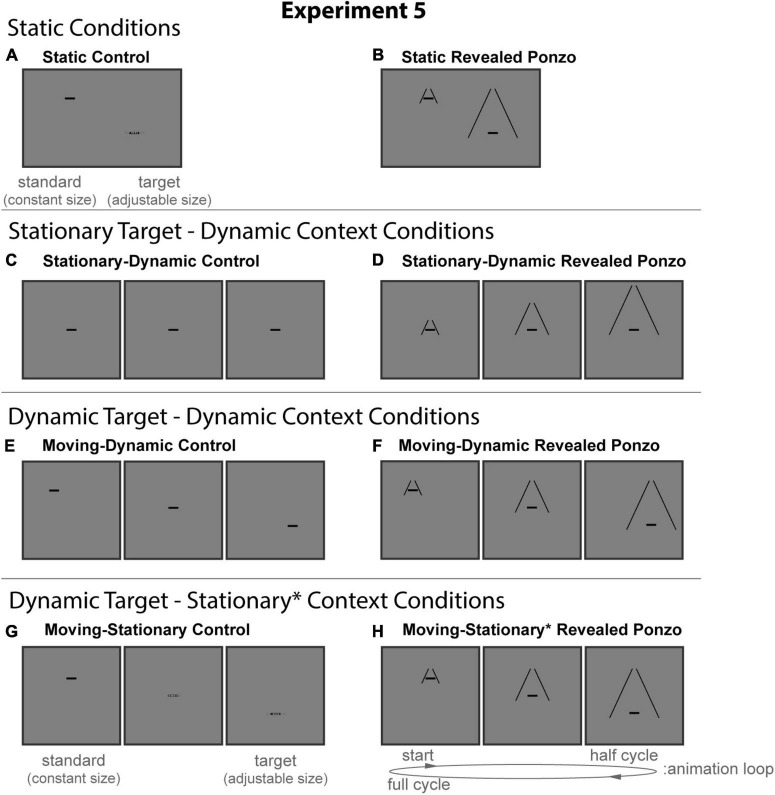
The eight conditions of Experiment 5. Columns represent the two distinct contexts [Control **(A,C,E,G)**, Revealed Ponzo **(B,D,F,H)**]. Rows represent the four configurations [Static **(A,B)**, Stationary-Dynamic **(C,D)**, Moving-Dynamic **(E,F)**, Moving-Stationary **(G,H)**]. For each condition, screenshots for the beginning (standard circle), middle, and end (target circle) frames are shown. The animation [as labeled in panel **(H)**] played in a continuous loop. In the figure, the horizontal line is the same physical size in all frames; during the experiment, the target (lower right line for Static conditions or the far-right frame for Dynamic conditions) was adjusted by the participant [see panels **(A,G)**]. Here, stimuli are cropped to display the central portion of the screen to save space. Supplementary Video 5 showing all of the dynamic conditions for Experiment 5 can be found in the Supplementary materials (best viewed in “loop” mode).

All stimuli were composed of black geometric shapes presented on a mid-gray background. Each participant was shown eight distinct conditions (Figure 10) formed from crossing three contexts (Control, Ponzo, and Revealed Ponzo) and four configurations (Static, Stationary-Dynamic, Moving-Dynamic, and Moving-Stationary). Supplementary Video 5 showing all dynamic conditions can be found in the Supplementary materials (best viewed in “loop” mode). Each participant completed eight trials per condition for a total of 64 trials. Participants were allowed to take self-timed breaks after every 20% of completed trials. Experiment 4 used the same method of adjustment task used in all other experiments. For the Static conditions, participants moved a computer mouse up and down to adjust the size of a target line to match the size of a nearby standard line (illustrated in Figure 10A). In the Dynamic conditions, participants adjusted the growth rate of a target line until they perceived it as not changing in size over time (illustrated in Figure 10G). There were no time limits; participants could take as much time as needed to make their response. Participants were allowed to freely view the display.

The static conditions of Experiment 5 (Figure 10, top row) include a masked variant of the classic Ponzo configurations in which the two comparison stimuli matched the endpoints of the corresponding dynamic conditions.

##### Static control

The static control condition (Figure 10A) was identical to the same condition in Experiments 3 and 4.

##### Static revealed Ponzo

The Static Revealed Ponzo condition (Figure 10B) was a mix of the Static Ponzo condition (Figure 6B) in Experiment 3 and the Static Masked Ponzo (Figure 8B) condition in Experiment 4. For the standard (upper left), only a limited extent of the surrounding lines (1.0° vertically) was visible; for the adjustable target (lower right), the full extent of the surrounding lines was visible. These configurations match the endpoints of the animation for the Dynamic Revealed Ponzo conditions, described below.

The stationary-dynamic conditions of Experiment 5 (Figure 10, second row) included a dynamically changing context surrounding a stationary target. We anticipate that illusion magnitudes would be weak for these conditions.

##### Stationary-dynamic control

The Stationary-Dynamic Control condition (Figure 10C) was identical to the same condition in Experiments 3 and 4.

##### Stationary-dynamic revealed Ponzo

All Revealed Ponzo conditions of Experiment 5 were similar to the corresponding Masked Ponzo condition of Experiments 3 and 4, with the exception that only the lower portion of the surrounding contextual lines were masked. In all Revealed Ponzo conditions, the mask occluded all contextual elements beyond 0.5° below the target line. The effect was that, over the course of the animation, the surrounding lines were “revealed” from top-to-bottom. The Stationary-Dynamic Ponzo condition (Figure 10D) was similar to the corresponding Masked Ponzo condition of Experiments 3 and 4, with the exception that the mask was only applied to the lower portion of the surrounding context.

The remaining conditions added diagonal or vertical translation of the target, in addition to the same context dynamics described for the above conditions. The moving target-dynamic context conditions of Experiment 5 (Figure 10, third row) included a moving target surrounded by a context that was both translating (obliquely) and dynamically changing eccentricity.

##### Moving-dynamic control

The Moving-Dynamic Control condition (Figure 10E) was identical to the same condition in Experiments 3 and 4.

##### Moving-dynamic revealed Ponzo

The Moving-Dynamic Revealed Ponzo condition (Figure 10F) was similar to the corresponding Masked Ponzo condition of Experiments 3 and 4, with the exception that the mask was only applied to the lower portion of the surrounding context.

The final two moving target-stationary context conditions of Experiment 5 (Figure 10, bottom row) included vertical target motion. As with similar conditions in Experiment 3 and 4, the context dynamically changed in terms of the eccentricity and length of the surrounding lines due to the masking procedure. Thus, while we maintain the same naming conventions as in other experiments, we denote this departure from a stationary context with an asterisk.

##### Moving-stationary control

The Moving-Stationary Control condition (Figure 10G) was identical to the same condition in Experiments 3 and 4.

##### Moving-stationary* revealed Ponzo

The Moving-Stationary* Revealed Ponzo condition (Figure 10H) was similar to the corresponding Masked Ponzo condition of Experiments 3 and 4, with the exception that the mask was only applied to the lower portion of the surrounding context.

#### Quantifying point of subjective equality and illusion magnitudes

Point of subjective equality and illusion magnitudes were computed using an identical procedure to that described for Experiment 1.

#### Statistical analysis

The statistical procedures were identical to those described for Experiment 1.

### Results and discussion

We compared average illusion magnitudes for the four conditions with a surrounding context for Experiment 5. If there were no illusory effects, we would expect illusion magnitudes to be zero. Means, confidence intervals, one-sample *t*-tests against zero for all conditions, and paired *t*-tests for all pairwise comparisons are summarized in Table 5. Individual and group-averaged illusion magnitudes are shown in Figure 9B.

We verified that illusion magnitudes differed across conditions using a repeated measures ANOVA. Mauchly’s test revealed a violation of sphericity, ε = 0.53, χ^2^(5) = 42.0, *p* < 0.001, and so we report the Greenhouse–Geisser correction. There was a significant main effect of condition, *F*(1.58,37.92) = 21.72, *p* < 0.001, $\eta_{p}^{2}$ = 0.48. Below, we focus on specific comparisons related to our *a priori* hypotheses using Bonferroni-corrected *post-hoc* tests.

The pattern of results for the Revealed Ponzo conditions of Experiment 5 was qualitatively similar to our previous published results for the Dynamic Ebbinghaus and Dynamic Corridor illusions (Mruczek et al., 2015, 2020) and Experiment 1. The Static Revealed Ponzo condition showed a significant illusory effect. The Stationary-Dynamic Revealed Ponzo condition, in which the target was stationary, led to a very weak illusory effect that was weaker than the Static Revealed Ponzo condition. Of particular interest is the Moving-Dynamic Revealed Ponzo condition, which combined target motion and a dynamically changing context. This configuration led to a robust illusion and for the first time in all of the configurations we have tested and similar to what we have observed for the Dynamic Ebbinghaus variations, resulted in a size illusion that was substantially larger than the magnitude than its static control condition (Table 5 and Figure 9B).

The Moving-Stationary* Revealed Ponzo condition, in which the target translated vertically between growing contextual lines, led to an illusion that was no different than the corresponding static condition. Although this condition is similar to the Moving-Dynamic Revealed Ponzo, the vertical translation of the target (across all experiments) tended to yield a weaker effect than the oblique translation. It may be an easier task to judge the size of a vertical target, for example by attending to the edges of the line.

Overall, the results of Experiment 5 suggest that the combination of target motion and a dynamically changing context can lead to enhanced size illusions, even for the size constancy Ponzo configuration.

## General discussion

The goal of this study was to explore the situations in which dynamic elements alter the magnitude of size constancy and size contrast illusions. In particular, we previously reported that the addition of dynamic elements (moving target) to the Corridor illusion (a size constancy illusion) can substantially *weaken* illusion magnitudes, whereas addition of dynamic elements (moving target, moving inducers, and growing/shrinking inducers) to the Ebbinghaus illusion (a size contrast illusion) can substantially *enhance* illusion magnitudes (Mruczek et al., 2020). Here, we tested the hypothesis that both classes of size illusions are enhanced in the presence of both a moving target and a dynamically changing contextual surround.

Experiments 1 and 2 show that the combination of a moving target and dynamically changing inducers (in terms of their size and eccentricity) greatly enhances the Ebbinghaus illusion. Indeed, the Moving-Dynamic Ebbinghaus illusion magnitude was double that of the familiar Static Ebbinghaus illusion. Dynamic inducers alone were not sufficient; the Stationary-Dynamic Ebbinghaus condition with a non-moving target did not produce a significant illusion. This is a highly robust pattern of results that has been replicated multiple times by us (Mruczek et al., 2015, 2020; Experiments 1 and 2) and others (Takao et al., 2021).

The current results also help place our previous findings (Mruczek et al., 2020) for a dynamic version of the Corridor illusion in perspective. The Dynamic Corridor in Mruczek et al. (2020) included an unchanging background with a moving target, which led to a significantly *weaker* illusion. In the current study, too, a moving target on an unchanging background (e.g., Moving-Stationary Corridor of Experiment 1 and Moving-Stationary Ponzo of Experiment 3) led to weak illusory effects. Adding translation of the entire stimulus (e.g., Moving-Dynamic Corridor of Experiment 1 and Moving-Dynamic Ponzo of Experiment 3), including the contextual lines providing linear perspective, also led to weak illusory effects. Thus, motion of the context was not sufficient to enhance illusion magnitudes. However, the pattern of results for the masked Corridor (Experiment 2), masked Ponzo (Experiment 3 and 4), and revealed Ponzo illusions (Experiment 5) show that a dynamically *changing* context led to strong illusory effects. In these dynamic illusions, the contextual lines changed size and/or eccentricity, in conjunction with target motion.

Thus, we observe a similar pattern of results for dynamic versions of the Ebbinghaus (size contrast) and Corridor and Ponzo (size constancy) illusions. Namely, the combination of (1) target motion and (2) a dynamically changing context led to enhanced illusions compared to when either of those dynamic components were separately present. One difference, however, is how strong the moving target-dynamic context illusions were relative to the corresponding “baseline” static conditions. As noted above, the Moving-Dynamic Ebbinghaus was twice the magnitude of the Static-Ebbinghaus illusion (Experiment 1). In contrast, the Moving-Dynamic Ponzo (Experiment 3) and Moving-Dynamic Masked Ponzo (Experiment 4) were equal to, but not stronger than, the corresponding static control condition. Only the Moving-Dynamic Revealed Ponzo (Experiment 5) yielded a pattern of results qualitatively similar to the Ebbinghaus illusion, with a substantially stronger illusion than the corresponding static control condition. One interpretation of this pattern of results is that the masked Ponzo (Experiment 3 and 4), with only short surrounding lines differing in eccentricity, greatly lessened the available depth cues. However, illusion magnitudes for the full Static Ponzo (8.6%) and the modified Static Masked Ponzo (8.0%) were actually quite similar. Thus, another possibility is related to the fact that the revealed Ponzo illusion included contextual lines that dynamically changed in size, extending as the target moved. The masking procedure for the revealed Ponzo may have induced some elements of size contrast into this size constancy illusion. This interpretation is consistent with the observation that, across all of our experiments, the dynamic conditions that led to an enhanced illusion magnitude relative to the corresponding static conditions included a dynamic context that changed in size.

The idea that “doubly dynamic” stimuli produce robust illusory percepts is not unique to size contrast, or even size illusions. While a drifting Gabor may induce a weak sense of stimulus motion opposite to the internal drift, the effect is substantially stronger when the Gabor globally translates (Tse and Hsieh, 2006; Shapiro et al., 2010; Lisi and Cavanagh, 2015). In variations of this illusion, a physical vertical translation can appear markedly oblique. Similarly, apparent motion of a stationary object induced by a moving background (Duncker, 1929) may be enhanced if the object itself is moving perpendicular to the background motion (Wallach et al., 1978). This effect is even stronger when the target is briefly flashed within a moving frame (Özkan et al., 2021; Cavanagh et al., 2022).

At the same time, dynamic motion does not always enhance illusory effects. Takao et al. (2021) generated novel dynamic variants of the Müller-Lyer and orientation contrast illusions. In both cases, a dynamically changing context surrounding a stationary target (what we call Stationary-Dynamic, here) led to no illusory effect. For the Müller-Lyer illusion, adding target motion revived the illusion to a degree, but the dynamic version was still much weaker than the static Müller-Lyer illusion. And for the orientation contrast illusion, the same manipulation did not revive the illusion at all. Takao et al. (2021) suggest that the moving dynamic Ebbinghaus is so robust because the change in inducer size causes a sense of looming and receding, leading to a change in the perceived depth of the image. A perceived change in depth could cause the stimulus to be interpreted as a 3D image, rather than a 2D image, changing the nature of the task for the participants from one about proximal features to distal features of the target (Todorović, 2002, 2020). When the target is also moving, it is bound to the inducers through common fate, causing a substantial change in perceived size. In our study, it is possible that the Moving-Dynamic Revealed Ponzo (Experiment 5) produced a similar alteration of perceived depth.

However, other observations from the current study and our past studies are not fully consistent with this explanation. First, in the Moving-Stationary Corridor condition (Experiment 1; see also Mruczek et al., 2020), a target moves along a background with clear depth cues from linear perspective. Yet only a weak illusion is observed, even when the stimulus and background translate together (Moving-Dynamic Corridor, Experiment 1). More directly, our previous work with the dynamic Ebbinghaus illusion showed that the Stationary-Dynamic Ebbinghaus is more effective when the central stimulus is jittered in its position, frame-by-frame. This manipulation does not alter putative looming/receding cues and does not obviously lead to better grouping of the target and inducers. It is, however, consistent with our proposal that reducing the precision with which the retinal image of the target can be represented over time leads to a stronger influence of contextual cues on perceived size (Mruczek et al., 2014, 2017a).

Of course, these hypotheses are not mutually exclusive, and it could be that both precision-based weighting of size cues and added/enhanced depth cues play a role in motion-based modulation of size illusions. There is certainly evidence that multiple underlying mechanisms contribute to the classic Ebbinghaus illusion (Rose and Bressan, 2002), such as contour integration (Jaeger and Klahs, 2015; Todorović and Jovanović, 2018), figural extent (Kirsch and Kunde, 2021), and size constancy (Doherty et al., 2010). Likewise, the same is true for the Corridor and Ponzo illusions (Yildiz et al., 2021). Future research will be necessary to determine if and how motion dynamics modulate the contribution of depth cues (e.g., linear perspective, looming/receding) and geometric cues (e.g., contour attraction and repulsion, spatial extent) to size perception. In addition to careful control of cues and instructions to better direct participants to the perceptual feature of interest (Todorović, 2002, 2020), an approach that we believe would be particularly fruitful for testing this would be to directly manipulate and measure perceptions of depth in these dynamic (and static) illusions.

## Data availability statement

The raw data supporting the conclusions of this article will be made available by the authors, without undue reservation.

## Ethics statement

The studies involving human participants were reviewed and approved by the Institutional Review Board of the College of the Holy Cross. The participants provided their written informed consent to participate in this study.

## Author contributions

All authors contributed to the conception and design of the study and drafted sections of the manuscript. RM, MF, and SK collected the data and performed the statistical analysis. RM and GC revised the manuscript and approved the submitted version.

## Supplementary material

Supplementary Video 1 (Video 1.MP4). Video of all dynamic conditions in Experiment 1. Panel labels match those of the corresponding manuscript Figure 2.

Supplementary Video 2 (Video 2.MP4). Video of all dynamic conditions in Experiment 2. Panel labels match those of the corresponding manuscript Figure 4.

Supplementary Video 3 (Video 3.MP4). Video of all dynamic conditions in Experiment 3. Panel labels match those of the corresponding manuscript Figure 6.

Supplementary Video 4 (Video 4.MP4). Video of all dynamic conditions in Experiment 4. Panel labels match those of the corresponding manuscript Figure 8.

Supplementary Video 5 (Video 5.MP4). Video of all dynamic conditions in Experiment 5. Panel labels match those of the corresponding manuscript Figure 10.
